# Relationship of transitional regulatory B and regulatory T cells and immunosuppressive drug doses in stable renal transplant recipients

**DOI:** 10.1002/iid3.473

**Published:** 2021-06-08

**Authors:** Eman H Ibrahim, Mostafa Aly, Christian Morath, Douaa M Sayed, Naruemol Ekpoom, Gerhard Opelz, Caner Süsal, Volker Daniel

**Affiliations:** ^1^ Transplantation Immunology, Institute of Immunology University Hospital Heidelberg Heidelberg Germany; ^2^ Clinical Pathology Department, South Egypt Cancer Institute Assiut University Assiut Egypt; ^3^ Department of Nephrology University Hospital Heidelberg Heidelberg Germany; ^4^ Nephrology Unit, Internal Medicine Department Assiut University Assiut Egypt

**Keywords:** blood, cell culture, Foxp3, IL10, immunoregulation, immunosuppressive drug doses, renal transplant recipients, transitional Bregs, Tregs

## Abstract

**Objectives:**

Regulatory B cells (Bregs) and T cells (Tregs) are thought to be involved in the regulation of graft acceptance in renal transplant recipients. However, mechanisms that affect Breg differentiation and interaction with Tregs are rather unclear.

**Methods:**

Using eight‐color‐fluorescence flow cytometry, Tregs and CD19+ CD24hiCD38hi Bregs were analyzed in whole blood samples of 80 stable kidney transplant recipients, 20 end‐stage renal disease (ESRD) patients and 32 healthy controls (HC). In addition, differentiation of Bregs and Tregs was studied in different micromilieus using cocultures with strongly enriched B‐lymphocytes and autologous peripheral blood mononuclear cells stimulated with CpG and phytohemagglutinin.

**Results:**

Bregs were higher in HC than in ESRD patients and lowest in transplant recipients. Bregs were higher early as compared to late posttransplant. Posttransplant, high Bregs were associated with higher glomerular filtration rate (GFR) and lower C‐reactive protein (CRP). Higher doses and blood levels of ciclosporine, tacrolimus, and mycophenolate mofetil as well as higher doses of steroids were not associated with low Bregs. In contrast, most Treg subsets were lower when blood levels of ciclosporine, tacrolimus, and mycophenolate mofetil were higher. Tregs were not associated with Bregs, GFR, CRP plasma levels, and occurrence of rejection or infection. In vitro, differentiation of Bregs was strongly dependent on T cell support and was blocked by excessive or lacking T‐cell help. Tregs were not associated with Breg numbers in vitro.

**Conclusion:**

Bregs appear to be insensitive to high doses of posttransplant immunosuppressive drugs. The protracted Breg decrease posttransplant might be caused by impaired T cell support attributable to immunosuppressive drugs.

## Graphical abstract

Regulatory B cells (Bregs) might be insensitive to high doses of posttransplant immunosuppressive drugs. The delayed Breg decrease posttransplant might be caused by impaired T cell support mediated by immunosuppressive drugs. Circulating regulatory T cells (Tregs) show an inverse relationship to transitional Bregs in the present study, suggesting that they do not induce each other

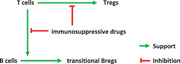

## 1 BACKGROUND

Regulatory B (Breg) cells are a heterogenous population of immunosuppressive cells that support immunological tolerance primarily through the production of interleukin 10 (IL10), IL35, transforming growth factor ß (TGFß), PDL1, Granzyme B, IDO, IgG4, and adenosine as reviewed by Rosser and Mauri.^1^, ^2^ IL10‐producing CD19+CD27+ memory B cells, CD19+CCD24hiCD27+ B10 cells,^3^, ^4^, ^5^, ^6^ and CD19+CD24hiCD27int plasmablasts,^7^ IL10‐and PDL1‐expressing CD19+CD24hiCD38hi immature B cells,^8^, ^9^, ^10^ IL10‐ and IgG4‐generating CD19+CD25hiCD71hi BR1 cells,^11^ granzyme B‐producing CD19+CD38+CD1d+ IgM+CD147+ GrB+^12^ and CD5+D43+CD86+CD147+ Breg cells,^13^ adenosine‐forming CD39+CD73+ B cells^14^ and TGF‐ and IDO‐producing induced Breg cells^15^ regulate and suppress T, B, NK, and dendritic cell responses during infection, autoimmunity, cancer, allergy, and pregnancy in mice and humans. There is evidence that Breg cells support good graft acceptance and tolerance induction in transplant recipients, as reviewed by several authors.^16^, ^17^, ^18^, ^19^ Moreover, certain Breg subsets were reported to induce and interact with regulatory T cells (Treg), as reviewed by Rosser and Mauri.^1^

Recently, we published a study of 10 renal transplant recipients who received a pretransplant infusion of ex‐vivo modified blood cells from the organ donor.^20^ Approximately 3 months posttransplant, the four patients who were treated with the highest dose of modified cells one week before the transplant operation showed an up to 68‐fold increase of CD19+CD24hiCD38hi Bregs, and virtually all of these Bregs produced IL10.^20^ Matched control transplant recipients receiving standard immunosuppression showed significantly lower Breg counts compared with these four patients.^20^ CD4+CD25+Foxp3+CD127− and CD4+CD25+CD127− Tregs were lowest on Day 30 after kidney transplantation, at the time of powerful immunosuppressive therapy.^20^

In the present study, we tried to find support for a potential clinically relevant role of Bregs in transplant recipients on standard immunosuppression. We focused on the total fraction of CD19+CD24hiCD38hi Bregs (total Bregs) and particularly on those CD19+CD24hiCD38hi Bregs with intracellular IL10 production (IL10+ Bregs). We compared Breg subsets of healthy controls (HC), patients with end‐stage renal disease (ESRD), and renal transplant recipients with each other and with various clinical parameters. In addition, we tried to elucidate whether Bregs are affected by high doses of posttransplant immunosuppressive drugs and whether Bregs increase during inflammation when their suppressive capacity is needed. In analogy to our previous study,^20^ we focused on patients during the early posttransplant period (median 116 days posttransplant), a time during which patients receive strong immunosuppression and a relationship of Bregs with the immunosuppressive protocol should be most apparent. In addition, we tried to find evidence for interactions of Bregs with Tregs.

In analogy to the micromilieus in vivo early and late posttransplant, we investigated Breg differentiation and Breg interaction with T lymphocytes in inflammatory and noninflammatory micromilieus in vitro. We hypothesize that the stimulated micromilieu might represent the situation immediately posttransplant, whereas the unstimulated micromilieu might correspond to the period late posttransplant when immunosuppressive drug doses are tapered and minimal immunosuppression is sufficient for maintaining graft quiescence and graft acceptance. Early posttransplant, up to 20% of patients experience biopsy confirmed acute rejections, as reported for renal transplant recipients receiving standard immunosuppression and/or treated with diverse immunomodulating protocols.^21^ We analyzed whether the results of our in vitro study supported the data obtained in vivo. To our knowledge, this is the first study describing the relationship of transitional Bregs, Tregs, and immunosuppressive drug doses in renal transplant recipients, and relevant in vitro experiments.

## 2 METHODS

### 2.1 Patients

Eighty‐one renal transplant recipients with stable good graft function and 20 ESRD dialysis patients from the Department of Nephrology, University of Heidelberg, were investigated. Thirty‐two healthy individuals, mostly staff members, served as controls. The study was approved by the Heidelberg ethical committee (S‐225/2014). Patients gave written informed consent for the tests performed within this study and the study was conducted in adherence to the Declaration of Helsinki. Table 1 summarizes the demographic data and the immunosuppressive protocol of 80 patients. In addition, one female patient transplanted in 1989 and identified with operational tolerance since 2006 was studied separately. Blood levels of mycophenolate mofetil (MMF), ciclosporine, tacrolimus, and everolimus were available according to immunosuppressive protocol from 14, 36, 36, and 2 patients, respectively. None of the ESRD patients and HC received immunosuppressive drugs. Three ESRD patients had received a kidney transplant more than 15 years ago. HC were younger and consisted of more female than male individuals compared to ESRD and transplant patients (Kruskal–Wallis test *p* < .001 and *p* = .023, respectively, Table 1). As reported by Blanco et al.,^22^ throughout life, the number of peripheral blood immature/transitional and naive B cells showed a similar profile to that of total B cells. The cells showed a peak at 1–11 months and a progressive decrease until adulthood. Subjects older than 18 years showed relatively stable numbers of both immature/transitional and naive B cells.^22^

**Table 1:** Demographic data of patients and healthy controls

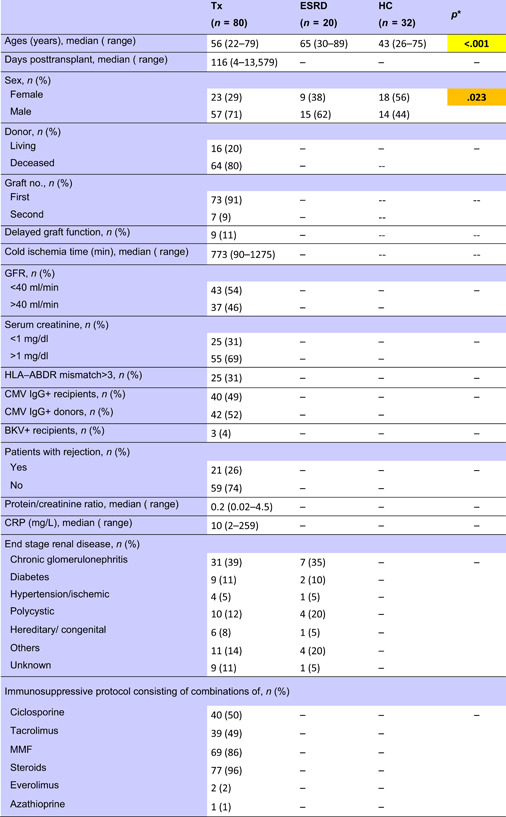

Abbreviations: CMV, cytomegalovirus; ESRD, end‐stage renal disease; GFR, glomerular filtration rate; HC, healthy control; HLA, human leukocyte antigen; MMF, mycophenolate mofetil; Tx, transplant recipients.

* Kruskal–Wallis test. *p* < .050 are given in bold with orange background and *p* < .010 are given in bold with yellow background.

#### 2.1.1 Determination of Bregs in the blood

Flow cytometric determinations were performed immediately after arrival of the blood samples in the lab. Five microliters of each fluorochrome‐labeled monoclonal antibody against CD45 (cat. 560178, BD), CD19 (cat. 564456, BD), CD38 (cat. 560677, BD), CD24 (cat. 555428, BD), CD127 (10 µl; cat. 560549; BD), CD4 (cat. 562970, BD), CD3 (cat. 563423, BD), CD8 (cat. 563919, BD), CD16 (cat. 557710, BD), and CD56 (cat. 566400, BD) were added to the tubes as recommended by the manufacturer, whereas antibodies against intracellular markers such as INFy (cat. 557643, BD), IL10 (cat. 564053, BD), TGFß1 (cat. 562339, BD), and Foxp3 (cat. 566526, BD) were not added until the permeabilization process had been performed. To each tube, 200 µl of whole blood was added. All tubes were vortexed briefly and incubated in the dark for 30 min at room temperature. Two milliliters of a 1:10 diluted Lyse solution from BD Biosciences was added to all tubes. Tubes were vortexed, incubated in the dark at room temperature for 10 min and centrifuged at 1300 rpm for 8 min. The supernatant was discarded, 1.5 ml phosphate‐buffered saline (PBS) was added and tubes were vortexed again briefly. The tubes were centrifuged at 1300 rpm for 8 min and the supernatant was discarded. Five hundred microliters of 1:10 diluted BD Permeabilizing II solution was added to the tubes. After 10 min incubation, 1.5 ml PBS was added. Tubes were vortexed briefly and subsequently centrifuged at 1300 rpm for 8 min. The supernatant was removed and discarded. Antibodies against the intracellular determinants Foxp3, IL10, IFNγ, and TGFβ were added to the pellets. After 30 min incubation in the dark at room temperature, 1.5 ml PBS was added. Tubes were vortexed again briefly and subsequently centrifuged at 1300 rpm for 8 min. The supernatant was removed and discarded. Finally, 100 µl PBS was added to the pellets and the cells were analyzed. All samples were evaluated with eight‐color fluorescence using the FACSCanto II triple‐laser flow cytometer (BD Biosciences). At least 100,000 events were analyzed in the initial FSC/SSC dot plot. The gating strategy is depicted in Figure 1.

Figure 1Gating strategy of total and IL10+CD19+CD24hiCD38hi Breg subsets, Tregs, and T cell proliferation. (A) Gating strategy for Bregs. Graph 1 shows all analyzed events; doublets are excluded (Graph 2). FSC versus SSC are dot‐plotted and lymphocytes are gated according to size (Graph 3). CD45 versus SSC dot plot permits focusing on lymphocytes and elimination of debris (Graph 4); B cells were identified using a CD19 versus SSC dot plot (Graph 5) and the CD24hiCD38hi Breg cluster was gated out of CD19+ lymphocytes (Graph 6). Graph 7 shows IL10+ Bregs gated from total Bregs. (B) Gating strategy for Tregs and their subsets. Graph 1 includes all the events analyzed in the tube. Graph 2 exhibits the exclusion of doublets. Graph 3 shows FSC versus SSC dot plot and gating of lymphocytes according to size, CD45 versus SSC dot plot permits elimination of debris and focuses on lymphocytes (Graph 4), CD25 versus CD4 dot plot allows the identification of CD4+CD25+ T lymphocytes (Graph 5), the Foxp3+CD127− cluster was gated out of CD4+CD25+ lymphocytes and identifies Tregs (Graph 6). In addition, Graphs 7–12 show Treg subsets gated out of total Tregs. (C) Gating strategy for T cell proliferation measured as proportion of blasts with low CFSE fluorescence (% CFSE low blasts). Graph 1 shows a dot plot of unstained cells in a cell culture incubated with cell culture medium for 3 days. Cells in the blast area R1 are depicted in red color. Graph 2 shows a cell culture incubated for 3 days with cell culture medium and CFSE. Lymphocytes are depicted in green color. Using a CFSE histogram, CFSE background staining of all cells in graph 2 was adjusted to less than 5% (M2 in graph 3). This CFSE gate was used for all further flow cytometric analyses when CFSE low blasts were measured in the blast area R2 and showed 1% CFSE low blasts for the 3‐day cell culture with cell culture medium and CFSE in the graphs 4 and 5. When cells were incubated with PHA and CFSE for 3 days, proportion of CFSE low blasts (proliferating T cells) increased to 50% (R2 in graph 6 and M2 in graph 7). Breg, regulatory B cell; CFSE, carboxyfluorescein succinimidyl ester; IL, interleukin; Treg, regulatory T cell

**Figure:**
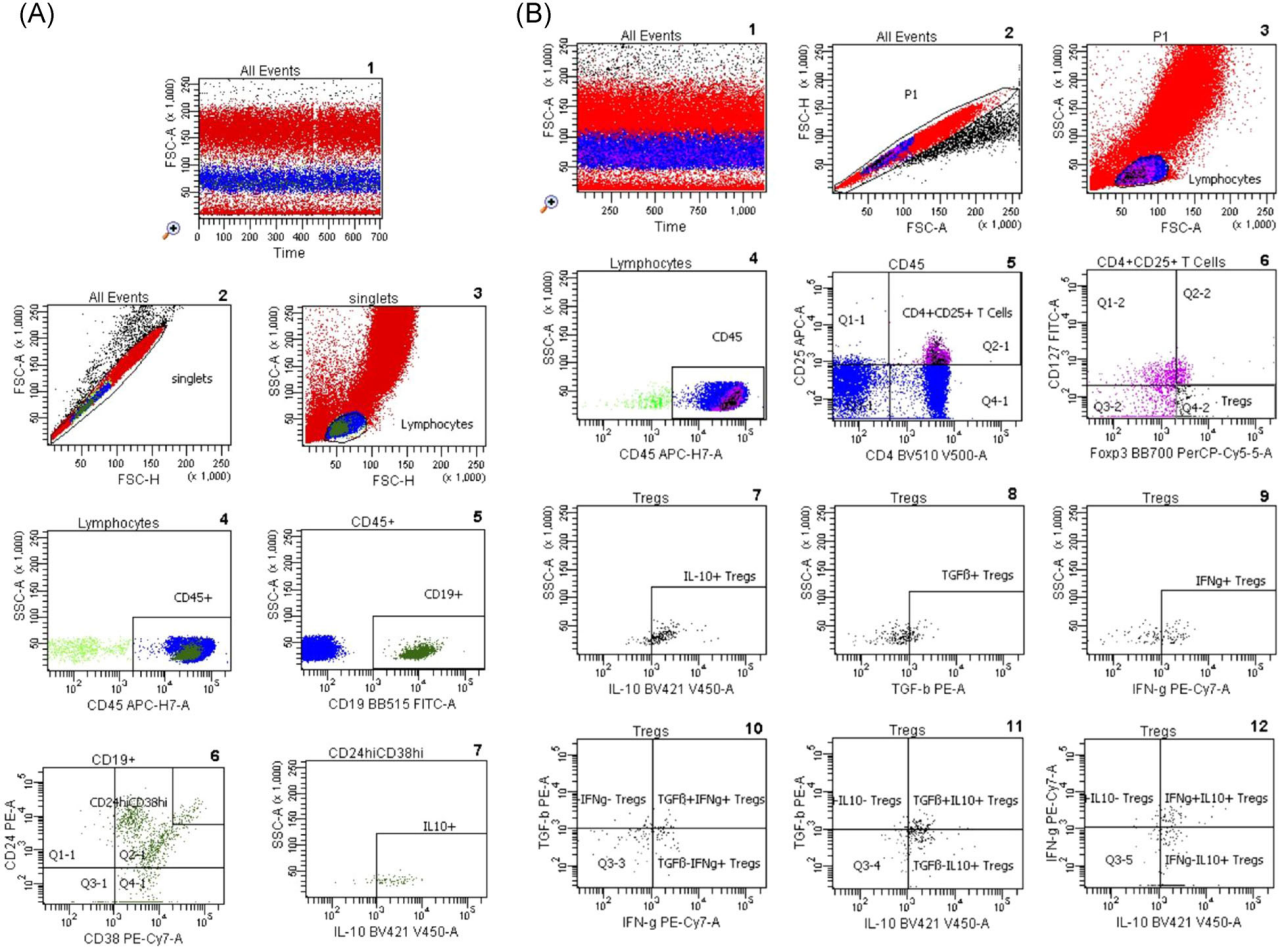

**Figure:**
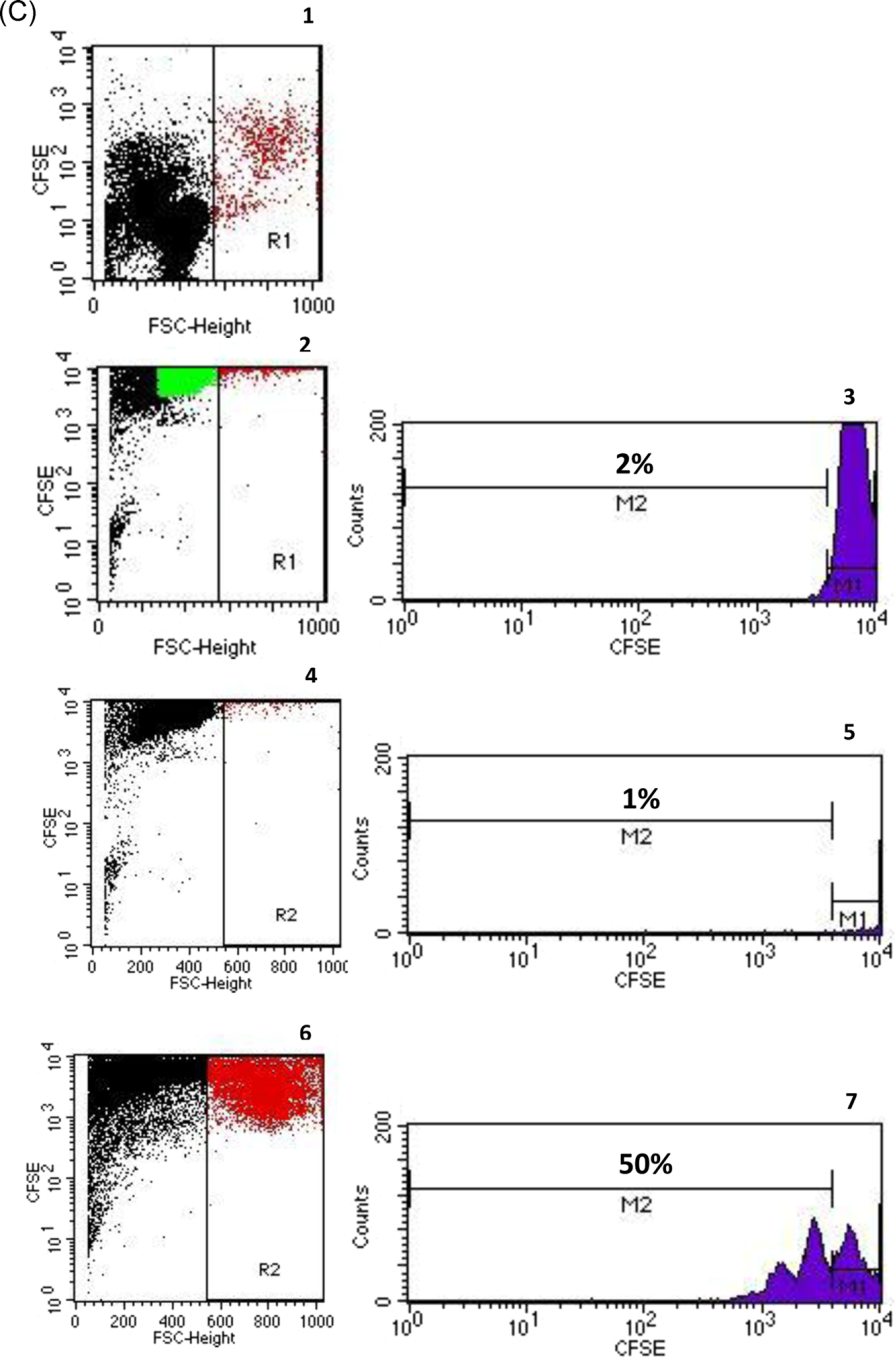

#### 2.1.2 Bregs in the cell cultures and cocultures

Twenty milliliters of heparinized blood was obtained from each of the 10 healthy volunteers. Peripheral blood mononuclear cells (PBMCs) were separated from heparinized whole blood using Ficoll density gradient centrifugation (Lymphodex; cat. 002041500; Innotrain). Half of the cells was stored frozen in dimethylsulfoxide‐containing freezing medium in liquid nitrogen for T cell stimulation, whereas the other half was used for enrichment of CD19+ B lymphocytes. CD19+ B lymphocytes were enriched using CD19‐coated Dynabeads (CD19panB, cat. 1143D; Invitrogen by Life Technologies) and antibody‐coated beads were removed from cell surface using DETACHaBEAD CD19 (cat. 12506D; Invitrogen by LIFE Technologies) according to the instructions of the manufacturer. Separated B lymphocytes were stimulated using the human TLR9 ligand class B CpG oligonucleotide, ODN 2006 (ODN 7909) (cat. Tlrl‐2006‐1, 1 mg; InvivoGen/Sigma‐Aldrich). One microliter (~2 µM) of ODN was added to separated B cells (2 × 10^6^ cells/ml) in 200 µl of cell culture medium containing RPMI 1640 (cat. 21875‐158, Invitrogen), Penicillin/Streptomycin (cat. 15140‐122) and l‐Glutamin (cat. 25030‐024, Gibco). Cells were incubated for 72 h in 96‐well cell culture plates, F bottom (cat. 655180, Greiner bio‐one). PBMCs were thawed and incubated for 72 h in the presence of B lymphocytes that had been cultivated previously with CpG for 72 h. Cocultures with phytohaemagglutinin (PHA; cat. 30852701; Remel Europe Ltd.) stimulated PBMCs were run in parallel. At the end of the cell culture period, cells were harvested, washed with DPBS (cat. 14190‐169; Invitrogen), and stained with fluorochrome labeled monoclonal antibodies. Treg panel: CD4 PerCP (cat. 345770, BD), CD25 APC (cat. 340907; BD), FoxP3 PE (cat. 560046; BD), CD127 FITC (cat. 11‐1278‐42; eBioscience); Breg panel: CD19 APC (cat. 345791; BD), CD24 PE (cat. 311106, Biolegend); CD27 FITC (cat. 302806; Biolegend), CD38 PerCP (cat. 303520; Biolegend), and the appropriate isotype controls for each monoclonal antibody. Treg and Breg proportions were determined using eight‐color fluorescence flow cytometry and a FACSCanto II (BD). In addition, proliferation of PBMCs was determined using carboxyfluorescein succinimidyl ester (CFSE; CellTrace™ CFSE cell proliferation kit (cat. C34554; Invitrogen by Thermo Fisher Scientific). Two microliters of CFSE were added to 1 ml PBMC suspension (2 × 10^6^ cells), PBMCs were incubated with PHA for 72 h and at the end of the cell culture lymphoblasts with low CFSE fluorescence were analyzed, representing the proportion of proliferated T cells in the cell culture. The gating strategy is depicted in Figure 1.

### 2.2 Statistical analysis

Descriptive statistics, Spearman's rank correlation test, Mann–Whitney *U* test, Kruskal–Wallis test and multiple regression analysis were performed using IBM SPSS statistics 25. We considered a *p* < .010 as significant and *p* values of .05–.01 as a weak association.

## 3 RESULTS

### 3.1 Bregs in HC, ESRD, and transplant patients

**Figure 2:**
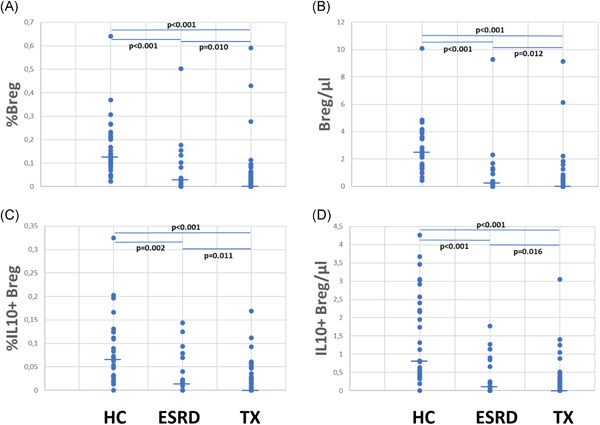
Total and IL10+CD19+CD24hiCD38hi regulatory B cell (Breg) subsets in stable transplant recipients, patients with end‐stage renal disease (ESRD) and healthy controls. Transplant recipients (Tx), patients with ESRD and healthy controls (HC) show different (A) relative (*p* < .001) and (B) absolute (*p* < .001) numbers of total CD19+CD24hiCD38hi, and (C) relative (*p* < .001) and (D) absolute (*p* < .001) numbers of IL10+CD19+CD24hiCD38hi Bregs (Kruskal–Wallis test). In addition, pairwise comparisons using Mann–Whitney *U* test are depicted. Horizontal bars represent medians. Relative cell numbers are related to CD45+ total lymphocytes

HC showed the highest relative and absolute Breg counts, ESRD patients exhibited lower Breg counts, and by far the lowest Breg counts were observed in transplant recipients (Figure 2).

**Figure 3:**
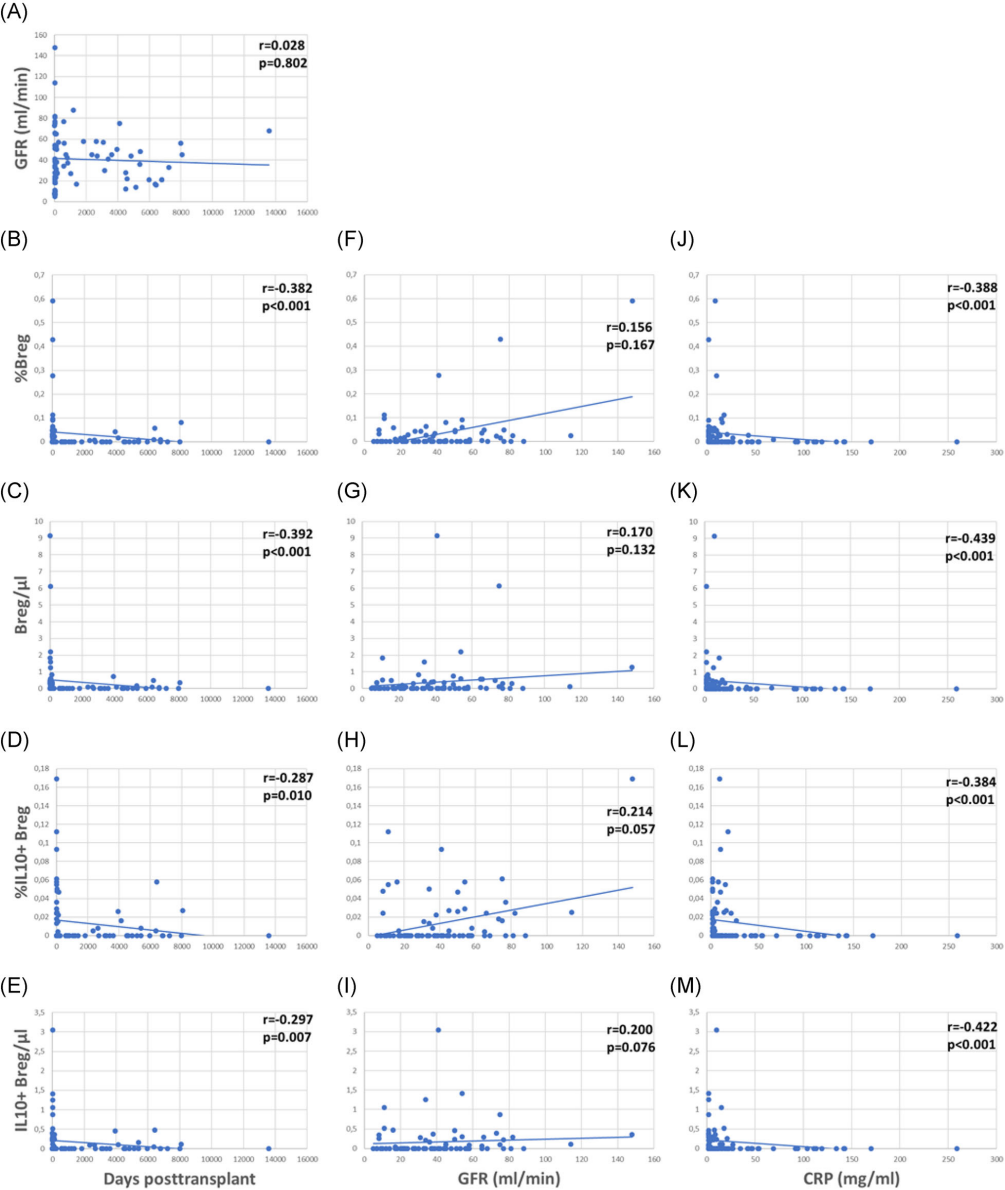
Total and IL10+CD19+CD24hiCD38hi regulatory B cells (Bregs), days posttransplant, glomerular filtration rate, and C‐reactive protein. Relative and absolute numbers of total and IL10+CD19+CD24hiCD38hi Breg subsets are associated with (B–E) days posttransplant and (J–M) C‐reactive protein (CRP) (Spearman's rank correlation test). There is no association of glomerular filtration rate (GFR) with (A) days posttransplant and only a weak trend of higher Bregs in patients with higher GFR (F–I)

Patients were studied 4–13,579 (median 116) days posttransplant (Figure 3A). Relative and absolute numbers of total and IL10+ Bregs were highest in patients early posttransplant and lower in patients tested later during follow up (Figure 3B–E). Forty‐five of the 80 (56%) patients were studied less than 1 year posttransplant and Bregs decreased during the first posttransplant year, but remained stable at a low level during the following years (Table A1).

The patients studied were selected to have good stable graft function without indication of acute rejection. Median glomerular filtration rate (GFR) was 37 ml/min (Figure 3A). GFR was not associated with days posttransplant, in other words there were patients with higher GFR early as well as later posttransplant (Figure 3A). There was only an insignificant trend of higher Bregs in patients with high GFR (Figure 3F–I). Higher Bregs were weakly associated with higher GFR during the first posttransplant year but not during the following years (Table A1). To determine whether the percentage of CD19+CD24hiCD38hi Bregs is independently associated with GFR in renal transplant recipients, we conducted a multiple multivariable regression analysis to account for the following potential confounders: Age, cold ischemia time, delayed graft function, HLA mismatches >3 (A, B, DR), CMV infections, BK‐nephropathy, rejections, and CRP. A significant positive correlation was shown. Relative numbers of CD19+CD24hiCD38hi Bregs were significantly associated with GFR (Table A2). GFR was not associated with absolute Breg counts (Table A2). Proteinuria was not related to Breg counts (Breg/µl *p* = .156, %Breg *p* = .164, IL10+ Breg/µl *p* = .247 and %IL10+ Breg *p* = .217, data not shown). High relative and absolute numbers of total and IL10+ Bregs were strongly associated with low C‐reactive protein (CRP) (Figure 3J–M) and this association was observed during the first as well as during the following posttransplant years (Table A1). These data argue against the presence of high Bregs during inflammation.

Patients without complications showed higher Breg numbers (Table A3) than those with previous infections and/or rejections during the postoperative course. At the time of testing, there was no difference in GFR (Table A3) or doses and blood levels of immunosuppressive drugs between the two patient groups (data not shown), confirming stable graft function in both patient groups at the time of investigation. However, patients with previous complications had lower proportions of CD4+ lymphocytes, higher percentages of CD56+CD16+ NK cells and slightly higher CRP blood levels (Table A3), suggesting persisting subliminal inflammation in these patients. The data do not support a presence of high Bregs during inflammation.

**Figure 4:**
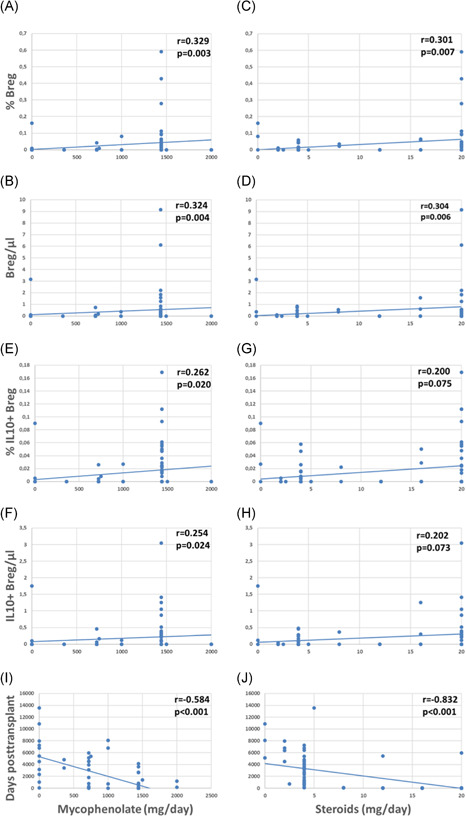
Total and IL10+CD19+CD24hiCD38hi regulatory B cells (Bregs), doses and blood levels of immunosuppressive drugs. (A–D) Higher relative and absolute counts of total Bregs are associated with higher daily doses of MMF and steroids (Spearman's rank correlation test) (E, F) and higher MMF doses with higher relative and absolute numbers of IL10+ Bregs, (G, H) whereas higher steroids doses show only a weak trend with higher IL10+ Bregs, (I, J)

Patients with higher daily drug doses of MMF and steroids showed higher relative and absolute numbers of total Bregs and weakly or borderline increased IL10+ Bregs as compared to patients receiving lower doses of these immunosuppressants (Figure 4A–H). MMF and steroids doses were tapered posttransplant (Figure 4I,J) and Bregs were associated with drug doses especially during the first postoperative year (Table A1). Higher tacrolimus blood levels were weakly associated with higher percentages of total Breg counts (*r* = .365; *p* = .034, data not shown) whereas daily doses of ciclosporine and tacrolimus as well as serum levels of MMF, ciclosporine and tacrolimus were not associated with total and IL10+ Breg counts (data not shown). When the two Breg subsets were analyzed in the context of induction therapy with basiliximab (*n* = 63), ATG (*n* = 11), or unknown antibodies (*n* = 7), there was no difference in Breg subsets among the patient subgroups (Kruskal–Wallis test, Breg/µl *p* = .882, %Breg *p* = .634, IL10+ Breg/µl *p* = .905 and %IL10 + Breg *p* = .777, data not shown). The seven patients with unknown induction therapy were transplanted in other countries and details with respect to induction therapy were not available. Altogether, these findings argue against the hypothesis that Bregs decrease rapidly in the presence of higher drug doses and higher blood levels of immunosuppressive drugs early posttransplant. The data rather suggest that Bregs are fairly insensitive to immunosuppressive drugs.

### 3.2 Tregs in HC, ESRD, and transplant patients

HC showed strong positive associations of different CD4+CD25+Foxp3+CD127− Treg subsets with total and high IL10+ Bregs, whereas these relationships were absent in ESRD patients and transplant recipients (Table 2). There was only a weak association of IL10+ Breg with IFNy−TGFß+ and IL10+TGFß+ Tregs in transplant recipients (Table 2).

**Table 2:** Correlation coefficients of associations of total Bregs and IL10+ Bregs with CD4+CD25+Foxp3+CD127− Treg subsets in the blood of 80 transplant recipients, 20 ESRD patients, and 32 healthy controls

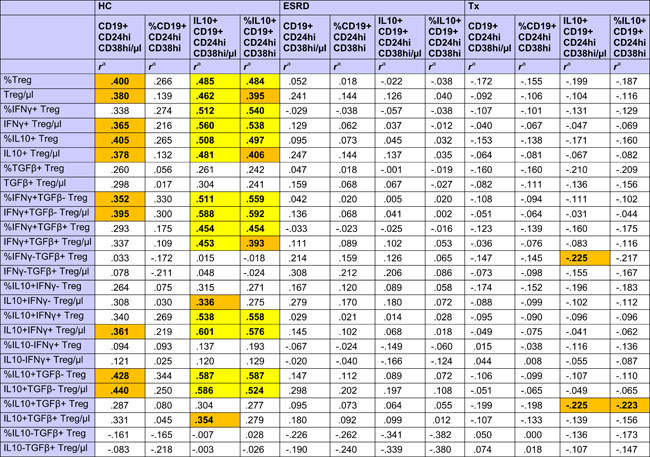

Abbreviations: Breg, regulatory B cell; ESRD, end‐stage renal disease; IFNγ, interferon γ; IL, interleukin; TGFβ, transforming growth β; Treg, regulatory T cell

^a^ *r* Values were calculated using Spearman's rank correlation test. *R* values of associations with a *p* < .050 are given in bold with orange background and of associations with a *p* < .010 in bold with yellow background.

**Figure A1:**
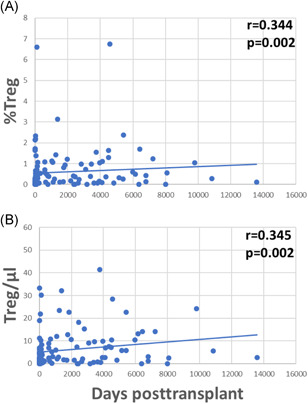
Regulatory T cells (Tregs) and days posttransplant. Absolute and relative numbers of CD4+CD25+Foxp3+CD127− Treg cells in the blood of 80 renal transplant recipients are higher late compared to early posttransplant (Spearman's rank correlation test)

With the exception of IL10−IFNy+ and IL10−TGFß+ Treg subpopulations, all other investigated Treg subsets were higher late as compared to early posttransplant (Table 3 and Figure A1). A possible explanation for this could be the influence of immunosuppressive drugs on Tregs. All Treg subsets were inversely associated with the daily dose of steroids (Table 3), whereas IL10−IFNy+, IFNy+TGFß+, and IL10−TGFß+ Treg subpopulations lacked a relationship with steroids. Interestingly, IL10‐IFNy+ and IL10‐TGFß+ Treg were the only Treg subsets that were associated with CRP plasma levels, showing an inverse association with the inflammation marker (IL10−TGFß+/µl *p* = .011; %IL10−TGFß+ *p* = .022; IL10−IFNy+/µl *p* = .026; %IL10−IFNy+ *p* = .085; data not shown). Most of the Treg subsets were lower when blood levels of ciclosporine, tacrolimus and in part also mycophenolate mofetil were higher (Table 3). Treg subsets were not associated with GFR (Table 3), application of bolus steroid doses as rejection therapy or occurrence of infections (Treg/µl *p* = .564 and *p* = .331, %Treg *p* = .496 and *p* = .153, data not shown). The data suggest that Treg subsets were inversely associated with steroids and other immunosuppressive drug doses but not with graft function, rejection episodes and infectious events, in contrast to Bregs which were associated with good transplant function, high steroids and high MMF, and low frequencies of rejections and infections, as indicated previously. When Tregs were analyzed in the context of induction therapy with basiliximab (*n* = 63), ATG (*n* = 11), or unknown antibodies (*n* = 7), there was no difference in Tregs among the patient subgroups (Kruskal–Wallis test, Treg/µl *p* = .112 and %Treg *p* = .429, data not shown).

**Table 3:** Associations of immunosuppressive drugs with Treg subsets in the blood of 80 transplant recipients

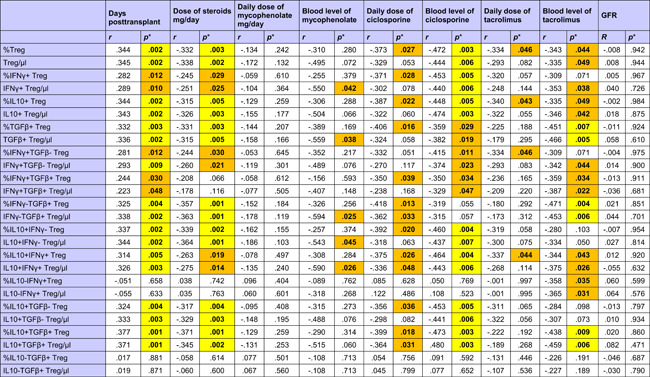

Abbreviations: GFR, glomerular filtration rate; IFNγ; interferon γ; IL, interleukin; TGFβ, transforming growth factor β; Treg, regulatory T cell.

* *p* Values were calculated using Spearman's rank correlation test. *p* < .050 are given in bold with orange background and *p* < .010 in bold with yellow background.

### 3.3 Bregs and Tregs in an operationally tolerant patient

In addition to the 80 patients on standard immunosuppression, one female patient transplanted in 1989 who had been identified to be operationally tolerant since 2006 and was off all immunosuppression was studied separately. The patient showed a GFR of 63 ml/min and a blood CRP level of 2 mg/l 10,850 days posttransplant. CD19+CD24hiCD38hi Breg counts were 3/µl and 0.16%, respectively, and IL10+CD19+CD24hiCD38hi Breg numbers were 2/µl and 0.09%, respectively, representing rather high but not the highest absolute and relative numbers within this entire group of transplant recipients. However, with respect to Breg numbers at the latest observation posttransplant, this patient showed the highest relative and absolute counts of CD19+CD24hiCD38hi and IL10+CD19+CD24hiCD38hi Bregs. Notably, this patient with operational tolerance 10,850 days posttransplant showed 6/µl or 0.3% of CD4+CD25+Foxp3+CD127− Tregs, which was rather similar to the median of 3/µl or 0.3% of CD4+CD25+Foxp3+CD127− Tregs found in all transplant recipients.

### 3.4 **Bregs, Tregs, IL10**+**CD3**+ **T cells, and T cell proliferation in cell cocultures**

Our data show that renal transplant recipients with an uncomplicated postoperative course (no infection, no rejection), better graft function and higher doses of immunosuppression showed the highest Breg counts in the blood. This observation suggests that Bregs differentiate more efficiently in a rather unstimulated than in an inflammatory micromilieu. We investigated this hypothesis using an in vitro model with enriched, CpG‐stimulated B cells added to cocultures with unstimulated or, alternatively, PHA‐stimulated autologous PBMC. Proportions of Bregs were calculated as percentages of total CD19+ lymphocytes determined in the cell cultures.

**Figure 5:**
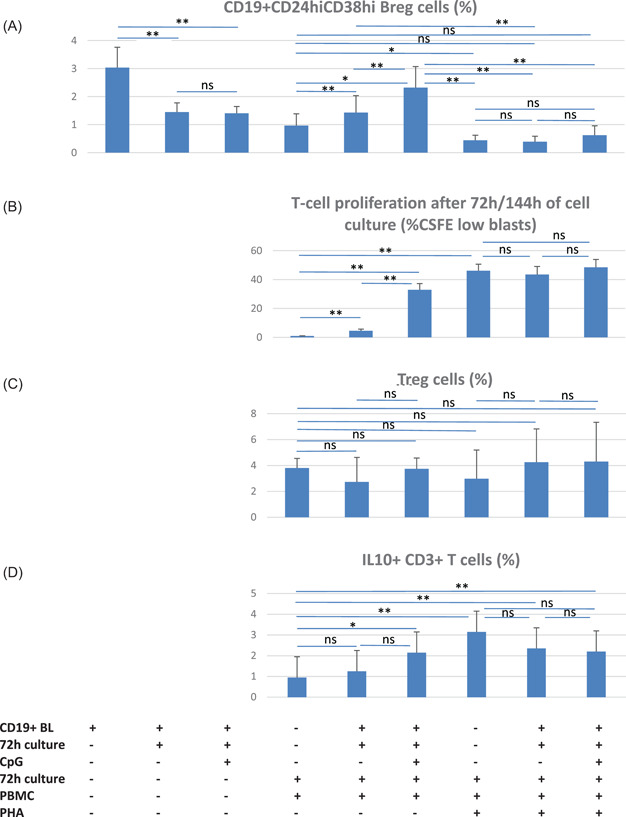
Proportions of CD19+CD24hiCD38hi regulatory B (Breg) cells in unstimulated and PHA‐stimulated cell cultures and cocultures. (A) Enriched CD19+ B lymphocytes separated from heparinized whole blood samples of 10 healthy controls consisted of 3% (median) CD19+CD24hiCD38hi Bregs. Proportion of Bregs decreased during 72‐h incubation in cell culture medium with or without CpG. When precultivated CD19+ B lymphocytes were added to autologous PBMCs and cocultured for another 72 h, Bregs recovered and Breg numbers were higher in cocultures with CpG precultivated B cells than in cocultures precultivated without CpG. In contrast, Bregs decreased strongly in all PHA‐stimulated cell cultures and cell cocultures

Freshly separated CD19+ lymphocytes of whole blood samples from 10 healthy individuals were enriched to 84% (median) CD19+ lymphocytes and 3% (median) CD19+CD24hiCD38hi Bregs (Figure 5). When enriched CD19+ lymphocytes were incubated in cell culture medium for 72 h in the presence or absence of CpG, Bregs decreased in both assays, however, Breg frequencies recovered when cultivated B cells were further cocultivated with autologous PBMC for another 72 h. Breg frequency in cocultures with CpG‐stimulated B cells was higher compared to the frequency in cocultures with B cells precultivated without CpG (Figure 5). The data indicate that activated B cells interact and differentiate stronger with autologous PBMC than unstimulated B cells.

Breg differentiation was inhibited in PHA‐stimulated cocultures. Thawed PBMC stimulated with PHA for 72 h contained less Bregs compared to PHA‐free cell cultures. Bregs did not increase when enriched B lymphocytes precultivated either with or without CpG were cocultivated with PHA‐stimulated PBMC (Figure 5). These data show that strong T cell stimulation in cocultures with resting or activated B cells impairs Breg differentiation.

The previous findings are supported by the observation that addition of B cells and Bregs to unstimulated T cells induces proliferation of T cells. Proliferation of T lymphocytes increased in 72 h PHA‐free cell cocultures with CpG‐free B lymphocytes and was stronger when CpG‐stimulated B cells were added (Figure 5). T cell proliferation increased strongly during 72 h stimulation with PHA and was similar in all three PHA‐stimulated cell cultures (with PHA alone, with CpG‐stimulated B cells, and with unstimulated B cells) (Figure 5).

The data suggest an interaction of resting T cells with resting or stimulated B cells that induces both T cell proliferation and Breg differentiation, whereas strong T cell proliferation in cocultures prevents Breg differentiation and decreases the number of Bregs in the cell culture. There was no evidence in the in vitro assays that Bregs inhibit T cell proliferation, presumably because of the imbalance of the small number of Bregs compared with the huge number of stimulated T cells.

Because both Bregs and T cells increased in PHA‐free cocultures, we speculated that Bregs might increase simultaneously with Tregs in the cell cocultures. However, the Treg proportion was similar in all cell cultures irrespective of PHA stimulation and addition of B lymphocytes precultivated in the presence or absence of CpG. Moreover, there was no correlation of Treg with Breg numbers in the cell cultures (Figure 5). The data suggest that increasing Breg numbers are not paralleled by increasing Treg numbers in the cell cultures.

IL10+CD3+ T cells represent another T‐cell subset with immunoregulatory phenotype. IL10+CD3+ T cells were similar in PHA‐free cell cultures with either medium or added unstimulated B lymphocytes, but were higher in PHA‐free cocultures with CpG‐stimulated B cells suggesting that moderate T cell stimulation by activated B cells induces IL10+CD3+ T cells in vitro (Figure 5).

This finding is substantiated by the observation that IL10+CD3+ T cells were higher in PHA‐stimulated cell cultures compared to PHA‐free cell cultures and in PHA‐stimulated cocultures with B lymphocytes precultivated with or without CpG (Figure 5). All three PHA‐stimulated cell cultures (PHA alone, with and without CpG) showed similar IL10+CD3+ T cell numbers. There was no association of IL10+CD3+ T cells with Breg numbers in the six cell cultures using Spearman's rank correlation (*p* = .508, *p* = .854, *p* = .150, *p* = .275, *p* = .298, and *p* = .226, data not shown).

The data suggest that IL10+CD3+ T lymphocytes increase in cell cultures with strongly stimulated T lymphocytes irrespective of Breg numbers and tend to increase in PHA‐free cell cocultures with added CpG‐stimulated Bregs.

## 4 DISCUSSION

Breg cells are able to modulate immune responses by suppressing the effector function of T cells, NK cells, plasma cells, macrophages and dendritic cells and, in addition, by inducing other regulatory cell subsets such as Tregs, as reviewed by Li et al.^19^ In clinical transplantation medicine, it is currently being investigated whether Bregs are capable of counter‐regulating immune responses against the graft.^19^

Contrary to our expectation, Breg numbers in the blood were found to be highest in healthy individuals, lower in ESRD patients, and lowest in transplant recipients. In search for an explanation for the low Breg numbers in transplant patients, one might speculate that previous but not current immunosuppressive therapies, in combination with immunosuppressive effects of the primary disease, uremia, and dialysis treatment, might contribute to a decrease of Breg numbers in these patients, and that these immunosuppressive influences might have a more chronic impact on circulating Bregs, resulting in slow Breg reduction in the circulation. Our data agree with reports of others describing higher total and/or IL10+ Breg numbers in HC compared to patients with nephropathy/dialysis^23^, ^24^, ^25^, ^26^ or transplant recipients,^25^, ^27^, ^28^, ^29^, ^30^, ^31^ and transplant recipients pretransplant as compared to posttransplant. Our data disagree with reports describing similar Breg numbers in ESRD patients and HC.^32^, ^33^, ^34^

The associations of higher Breg counts with higher daily doses of steroids and MMF, higher GFR and lower CRP could be suspected to be due to the observation that Breg counts are highest during the early posttransplant period when patients receive the highest doses of immunosuppressive drugs. However, daily doses of ciclosporine and tacrolimus and blood levels of MMF, ciclosporine and tacrolimus were not associated with Breg counts. Moreover, because Breg counts were significantly higher in patients early posttransplant compared with patients late posttransplant, we may assume that stepwise tapering of the immunosuppressive drugs was not paralleled by a stepwise increase of Breg counts in the blood. It appears altogether that peripheral Breg counts are not acutely reduced by postoperatively administered high doses of immunosuppressive drugs. Our observations agree with those of Latorre et al. showing that transplant recipients display lower numbers of transitional B cells in the blood than HC, and that Breg numbers correlate with long‐term therapeutic graft survival irrespective of CNI or mTOR inhibitor treatment.^27^ Our data are in line with those of Tebbe et al. describing low Bregs in patients with low GFR^28^ and those of Piloni et al. who published that transitional B cells might participate in long‐term lung graft acceptance mechanisms and that Breg blood cell counts were reduced during high MMF treatment but not during high ciclosporine, tacrolimus, azathioprine, rapamycin or prednisone therapy.^35^ In contrast to Piloni et al.,^35^ in our cohort of renal transplant recipients high Bregs were associated with high MMF drug doses. Conceivably, the association of Bregs with MMF dose changes with time, perhaps especially in patients who received a lung graft as studied by Piloni et al.^35^

One might speculate that higher Bregs downregulate cytotoxic effector cells and maintain low levels of these cells, especially during the early posttransplant period when other immunoregulatory subsets such as Treg cells are low and their differentiation is blocked by strong immunosuppressive treatment. Treg subsets are inversely associated with ciclosporine and tacrolimus blood levels as shown in Table 3 and this strong relationship explains the missing association of Treg and Breg blood counts and the negative association of IL10+, TGFß+, and IFNy−TGFß+ Treg subsets with IL10+ Bregs in renal transplant recipients shown in Table 2. In HC, but not in ESRD patients and kidney transplant recipients, Bregs were positively associated with Tregs and this observation supports our hypothesis that Bregs appear to be more resistant to immunosuppressive impacts than Tregs. Other authors analyzed 1106 Treg/Breg cell determinations in 117 lung transplant recipients and were also unable to find an association of Bregs with Tregs posttransplant.^35^ In addition to IL10+ and TGFß+ Tregs, we analyzed IFNy+ Treg. IFNy+ Tregs have been shown to modulate immune responses in vitro and were associated with clinical events in renal transplant recipients.^36^, ^37^, ^38^

It appears that Bregs and Tregs act synergistically in HC, forming a balance with cytotoxic effector cells, whereas in ESRD and renal transplant recipients this balance is missing, presumably due to the composite immunosuppressive influence of primary disease, uremia, dialysis therapy, and immunosuppressive drugs, as well as the greater susceptibility of Tregs to immunosuppressants compared to Bregs. We believe it is conceivable that in ESRD and renal transplant recipients predominantly Bregs, and not Tregs, maintain low cytotoxic responses of the immune system. This is in line with the observation that in operationally tolerant transplant recipients peripheral blood B cells were increased, particularly those showing the phenotype of transitional B cells.^39^, ^40^ We were able to investigate a patient transplanted 31 years ago who demonstrated operational tolerance during the last 14 years, showing excellent graft function and higher total as well as higher IL10+ Bregs in the absence of immunosuppressive treatment. The Tregs in this patient were in the range of transplant recipients with standard immunosuppression late posttransplant. We hypothesize that Bregs differentiate in this operationally tolerant patient due to normal T cell support.

The dependence of Bregs on T cell help is confirmed by our in vitro findings. Strongly enriched CD19+ B cells did not differentiate to Bregs in response to CpG stimulation, however, when cocultivated with autologous PBMC, differentiation to Bregs was enhanced, confirming that mutual interaction of B and T cells is essential for Breg differentiation, as reviewed by Wang et al. and as shown by Hong et al. for other Breg subsets such as CD19+CD25high Bregs.^41^, ^42^, ^43^ Excessive T cell stimulation completely inhibits Breg differentiation in the cocultures, presumably because strongly proliferating T cells consume all the cytokines that are essential for Breg differentiation. We speculate that the strong immunosuppressive therapy immediately posttransplant particularly inhibits T cells and thereby decreases the T cell support for Breg differentiation, whereas uremia causes premature ageing of the T‐cell compartment in ESRD patients^44^ resulting in reduced efficiency of vaccination,^45^ an enhanced susceptibility for infectious diseases^46^ and an enhanced risk for developing autoimmune diseases and tumors.^47^ Bregs themselves might be insensitive to immunosuppressants. In vitro, an increase of T cell activation and cytokines in the micromilieu appears to induce a Breg increase. Bregs show an indirect response to immunosuppressive drugs and increase in a moderate inflammatory micromilieu but not in a strongly inflammatory micromilieu. The moderate inflammatory micromilieu supports, in addition, differentiation of other cells with immunoregulatory phenotype, such as IL10+ T cells, that might act synergistically with IL10+ Bregs and might contribute to good graft acceptance in the transplant patients. Treg differentiation was not paralleled by Breg differentiation in the different coculture experiments, suggesting that transitional CD19+CD24hiCD38hi Bregs and CD4+CD25+CD127−Foxp3+ Tregs might not induce each other in this experimental setting, presumably due to the need of different cytokines for Treg and Breg differentiation. This conclusion agrees with the observation in our transplant recipients. Others reported that CD19+CD24hiCD38hi Bregs maintain regulatory T cells while limiting T helper 1 (Th1) and Th17 differentiation.^10^ CD19+CD24hiCD38hi Bregs obtained from HC‐inhibited naïve T cell differentiation into Th1 and Th17 cells and converted CD4+CD25− T cells into regulatory T cells, in part through the production of IL10. In contrast, CD19+CD24hiCD38hi B cells from patients with rheumatoid arthritis failed to convert CD4+CD25− T cells into functionally suppressive Tregs or to curb Th17 development; however, they maintained the capacity to inhibit Th1 cell differentiation.^10^ Interaction of Bregs with Tregs depends on the micromilieu in vitro and in vivo. As reviewed by Wang et al.,^42^ recent studies indicate that Breg cell populations are low under physiological conditions but increase substantially in both human patients and murine models of chronic inflammatory diseases, autoimmune diseases, infection, transplantation, and cancer. However, as demonstrated in our in vitro experiments, lack of or strong T cell stimulation inhibits Breg differentiation and this might have clinical relevance when occurring in patients.

### 4.1 Limitations

We studied clinically stable transplant recipients without acute rejection or infection in whom high Bregs were associated with high GFR, low CRP, and high drug doses. Patients during rejection or with other inflammatory events such as acute infections might show different Breg patterns, as recently described for kidney transplant recipients with antibody‐mediated rejection.^48^ The Breg pattern of our patients represents a well‐balanced immune system. During early inflammation, Bregs might increase moderately or remain stable high because they would be needed to downregulate the acute immune response. In contrast, low Breg counts in patients with high CRP levels confirm our in vitro finding of low Breg numbers in a strongly inflammatory micromilieu.

Another limitation might be that we studied Breg dynamics in drug‐free cell cultures with cells from healthy individuals. Our in vitro findings indicate that Breg increases are only feasible in cooperation with other cells, particularly T lymphocytes that stimulate Breg differentiation and this mechanism of Breg induction might operate in vitro and in vivo and might impact Breg differentiation in healthy individuals as well as transplant patients receiving immunosuppressive drugs.

### 4.2 Conclusion

**Figure A2:**
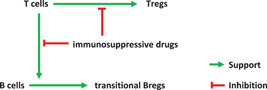
Relationship of transitional regulatory B (Breg) and regulatory T (Treg) cells and immunosuppressive drug doses in stable renal transplant recipients. Bregs might be insensitive to high doses of immunosuppressive drugs posttransplant. The delayed Breg decrease posttransplant might be caused by impaired T cell support mediated by immunosuppressive drugs. Circulating Tregs show an inverse relationship to transitional Bregs in the present study suggesting that they do not induce each other

In conclusion, the results of this study disprove our own hypothesis that Bregs are high during inflammation and low during strong immunosuppressive therapy. It appears that Breg numbers are not acutely downregulated by high doses and high blood levels of immunosuppressive drugs. The protracted Breg decrease posttransplant might be caused by impaired T cell support mediated by immunosuppressive drugs (Figure A2). A patient with operational tolerance and high Bregs studied in addition supports this finding. Circulating Tregs show an inverse relationship to transitional Bregs in the present study, suggesting that they do not induce each other. The results of the present study increase our knowledge of Breg differentiation and Breg/Treg interaction in kidney transplant recipients and might be useful for monitoring of Bregs and Tregs in patients receiving immunosuppressive drugs.

## CONFLICT OF INTERESTS

Christian Morath and Gerhard Opelz together with the University of Heidelberg, are cofounders of TolerogenixX GmbH, a biotechnology company that holds licenses for modified immune cell (MIC) treatment. Gerhard Opelz holds a patent for MIC treatment (“Immunosuppressive blood cells and methods of producing the same.” Patent no. WO 2010/000730, EP 2318020). Christian Morath, Volker Daniel, and Caner Süsal filed a patent application for MIC treatment (“MIC therapy for specific immunosuppression in transplantation.” Patent no. PCT/EP2019/062857).

## AUTHOR CONTRIBUTIONS

Volker Daniel, Eman H. Ibrahim, and Mostafa Aly designed the study, compilation and statistical analysis of the data. Eman H. Ibrahim performed all experiments of this study and assisted in writing the manuscript. Volker Daniel wrote the manuscript. Gerhard Opelz made substantial contributions to conception and design of the study and revised the manuscript critically. Mostafa Aly, Douaa M Sayed, Naruemol Ekpoom, Caner Süsal, and Christian Morath have been involved in important intellectual content. Christian Morath treated the patients. All authors have given final approval of the manuscript to be published.

## Data Availability

The datasets used and/or analyzed during the current study are available from the corresponding author on reasonable request.

## APPENDIX A

See Tables A1, A2, and A3

**Table A1:** Associations of total and IL10+ Bregs with clinical parameters

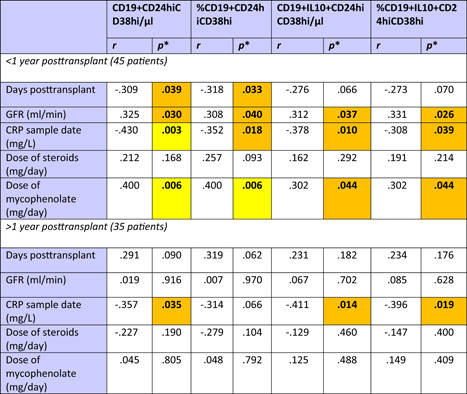

Abbreviations: Breg, regulatory B cells; CRP, C‐reactive protein; GFR, glomerular filtration rate; IL10, interleukin 10.

* Spearman's rank correlation test. *p* < .050 are given in bold with orange background and *p* < .010 in bold with yellow background.

**Table A2:** Regression analysis including Bregs and clinical parameters of 79 transplant recipients on standard immunosuppression^a^

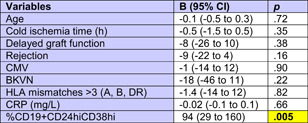

Abbreviations: BKVN, BK virus nephropathy; Breg, regulatory B cells; CI, confidence indetval; CMV, cytomegalovirus; CRP, C‐reactive protein; HLA, human leukocyte antigen.

^a^ The total of clinical data was available only from 79 renal transplant recipients.

**Table A3:** Associations of total and IL10+ Bregs with occurrence of infections and rejections posttransplant

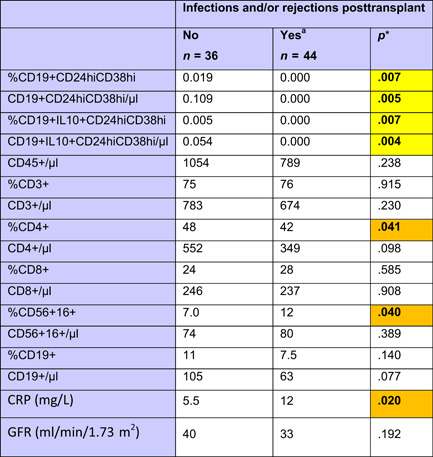

*Note*: All data are given as median. Relative cell numbers are related to CD45+ total lymphocytes.

Abbreviations: Breg, regulatory B cells; CRP, C‐reactive protein; GFR, glomerular filtration rate; IL10, interleukin 10.

^a^ Rejections posttransplant were assessed based on pulse dose steroids administrations and transplant biopsies. Eight patients were Inf+rej‐, 26 patients inf‐rej+ and 10 patients inf+rej+.

* Mann–Whitney *U* test. *p* < .050 are given in bold with orange background and *p* < .010 in bold with yellow background.
